# Neurophysiological evidence of motor preparation dysfunction to inner speech in schizophrenia

**DOI:** 10.1192/j.eurpsy.2024.516

**Published:** 2024-08-27

**Authors:** L. K.-H. Chung, A. W. Harris, O. Griffiths, B. N. Jack, M. E. Le Pelley, K. M. Spencer, A. R. Barreiros, A. W. Harrison, N. Han, S. Libesman, D. Pearson, R. B. Elijah, S. S.-M. Chan, G. H.-C. Chong, S. H.-W. So, T. J. Whitford

**Affiliations:** ^1^School of Psychology, University of New South Wales (UNSW Sydney), Sydney, Australia; ^2^Department of Psychology, The Chinese University of Hong Kong, Hong Kong, Hong Kong; ^3^Sydney Medical School, University of Sydney; ^4^Brain Dynamics Centre, Westmead Institute for Medical Research, Sydney; ^5^School of Psychological Sciences, University of Newcastle, Callaghan; ^6^Research School of Psychology, Australian National University, Canberra, Australia; ^7^Research Service, Veterans Affairs Boston Healthcare System; ^8^Department of Psychiatry, Harvard Medical School, Boston, United States; ^9^NHMRC Clinical Trials Centre; ^10^School of Psychology, University of Sydney, Sydney, Australia; ^11^Department of Psychiatry, The Chinese University of Hong Kong; ^12^Clinical Psychology Service, Kwai Chung Hospital, Hong Kong, Hong Kong

## Abstract

**Introduction:**

Auditory verbal hallucinations (AVHs) in schizophrenia have been suggested to arise from failure of corollary discharge mechanisms to correctly predict and suppress self-initiated inner speech. However, it is unclear whether such dysfunction is related to motor preparation of inner speech during which sensorimotor predictions are formed. The contingent negative variation (CNV) is a slow-going negative event-related potential that occurs prior to executing an action. A recent meta-analysis has revealed a large effect for CNV blunting in schizophrenia. Given that inner speech, similar to overt speech, has been shown to be preceded by a CNV, the present study tested the notion that AVHs are associated with inner speech-specific motor preparation deficits.

**Objectives:**

The present study aimed to provide a useful framework for directly testing the long-held idea that AVHs may be related to inner speech-specific CNV blunting in patients with schizophrenia. This may hold promise for a reliable biomarker of AVHs.

**Methods:**

Hallucinating (n=52) and non-hallucinating (n=45) patients with schizophrenia, along with matched healthy controls (n=42), participated in a novel electroencephalographic (EEG) paradigm. In the Active condition, they were asked to imagine a single phoneme at a cue moment while, precisely at the same time, being presented with an auditory probe. In the Passive condition, they were asked to passively listen to the auditory probes. The amplitude of the CNV preceding the production of inner speech was examined.

**Results:**

Healthy controls showed a larger CNV amplitude (*p* = .002, *d* = .50) in the Active compared to the Passive condition, replicating previous results of a CNV preceding inner speech. However, both patient groups did not show a difference between the two conditions (*p* > .05). Importantly, a repeated measure ANOVA revealed a significant interaction effect (*p* = .007, η_p_^2^ = .05). Follow-up contrasts showed that healthy controls exhibited a larger CNV amplitude in the Active condition than both the hallucinating (*p* = .013, *d* = .52) and non-hallucinating patients *(p* < .001, *d* = .88). No difference was found between the two patient groups *(p* = .320, *d* = .20).

**Conclusions:**

The results indicated that motor preparation of inner speech in schizophrenia was disrupted. While the production of inner speech resulted in a larger CNV than passive listening in healthy controls, which was indicative of the involvement of motor planning, patients exhibited markedly blunted motor preparatory activity to inner speech. This may reflect dysfunction in the formation of corollary discharges. Interestingly, the deficits did not differ between hallucinating and non-hallucinating patients. Future work is needed to elucidate the specificity of inner speech-specific motor preparation deficits with AVHs. Overall, this study provides evidence in support of atypical inner speech monitoring in schizophrenia.

**Disclosure of Interest:**

None Declared

